# Two Lithuanian Cases of Classical Galactosemia with a Literature Review: A Novel *GALT* Gene Mutation Identified

**DOI:** 10.3390/medicina56110559

**Published:** 2020-10-25

**Authors:** Rūta Rokaitė, Rasa Traberg, Mindaugas Dženkaitis, Rūta Kučinskienė, Liutauras Labanauskas

**Affiliations:** 1 Department of Pediatrics, Medical Academy, Lithuanian University of Health Sciences, LT 44307 Kaunas, Lithuania; rutadrk@gmail.com (P.K.); liutaurui.l@gmail.com (L.L.); 2 Department of Genetics and Molecular Medicine, Medical Academy, Lithuanian University of Health Sciences, LT 44307 Kaunas, Lithuania; rasa.traberg@gmail.com; 3 School of Biological Sciences, University of Edinburgh, Edinburgh EH9 3FF, UK; mindaugasdzenkaitis@gmail.com

**Keywords:** galactosemia, GALT, galactose-1-phosphate uridylyltransferase, newborn

## Abstract

Galactosemia is a rare autosomal recessive genetic disorder that causes impaired metabolism of the carbohydrate galactose. This leads to severe liver and kidney insufficiency, central nervous system damage and long-term complications in newborns. We present two clinical cases of classical galactosemia diagnosed at the Lithuanian University of Health Sciences (LUHS) Kaunas Clinics hospital and we compare these cases in terms of clinical symptoms and genetic variation in the *GALT* gene. The main clinical symptoms were jaundice and hepatomegaly, significant weight loss, and lethargy. The clinical presentation of the disease in Patient 1 was more severe than that in Patient 2 due to liver failure and *E. coli*-induced sepsis. A novel, likely pathogenic *GALT* variant NM_000155.4:c.305T>C (p.Leu102Pro) was identified and we believe it could be responsible for a more severe course of the disease, although further study is needed to confirm this. It is very important to suspect and diagnose galactosemia as early in its course as possible, and introduce lactose-free formula into the patient’s diet. Wide-scale newborn screening and genetic testing are particularly crucial for the early detection of the disease.

## 1. Introduction

Galactosemia is a rare autosomal recessive genetic disorder, which causes impaired metabolism of the carbohydrate galactose [1]. Galactose is mostly produced following the breakdown of lactose, which is the main carbohydrate in breast milk, which in itself makes up the majority of a newborn’s diet. Even though the main source of both lactose and galactose is milk, they can also be found in other foods. In the human body, lactose is broken down into glucose and galactose (by the enzyme lactase). The latter is then further broken down by the enzymes galactokinase (GALK), galactose-1-phosphate uridylyltransferase (GALT) and UDP-glucose 4-epimerase (GALE) [2]. In the absence of any of these enzymes, toxic breakdown products of galactose accumulate in the body, which results in damage to the liver, kidneys, eyes, central nervous system, and inhibits the child’s development. Depending on which enzyme is missing (i.e., which gene mutation had occurred), the symptoms of galactosemia can vary in their severity [3].

Classical galactosemia is caused by the lack of GALT, which can result from the individual being homozygous or compound heterozygous for a pathogenic variant of the *GALT* gene [3]. The *GALT* gene is made up of 11 exons, and is ~4 kb in length. At the time of writing, more than 300 known variants exist for this gene [3,4]. Most of these are missense and nonsense mutations, splice site variations, or intragenic deletions [4].

The classical type of galactosemia is diagnosed more often than the other types [5,6]. This form of galactosemia starts to show symptoms in the neonatal stage of life, when galactose first enters the body. Galactose-1-phosphate, galactitol, as well as other toxic metabolites, begin to accumulate in the body and are responsible for the resulting damage to internal organs [1]. Due to acute toxicity of the compounds, newborns develop jaundice, hepatosplenomegaly, liver failure, food intolerance, hypoglycemia, renal tubular dysfunction, muscle hypotonia, sepsis and even die [7].

The prevalence of classical galactosemia depends on ethnic background. This disease is more widespread among individuals of European ancestry than those of Asian ancestry—its prevalence ranging from 1:23 500–1:44 000 in the UK, 1:42 000 in Lithuania and 1:50 000 in the US to 1:400 000 in Taiwan and 1:700 000 in Japan [5,8,9]. In many countries, including Lithuania, this disease is part of the State Newborn Screening (NBS) programs [6,9]. All newborns in Lithuania have been screened for galactosemia since 2015. To the best of our knowledge, from 2015 to 2019 there have been 3 diagnosed cases of classical galactosemia as well as 2 cases of Duarte galactosemia in Lithuania (no specific treatment is given for those affected by Duarte galactosemia) [9].

In this article, we describe two clinical cases of classical galactosemia, which were diagnosed at the LUHS (Lithuanian University of Health Sciences) Kaunas Clinics hospital. Both patients were born at two separate small town hospitals located in the same region of the country.

## 2. Case Report

Patient 1 was a female neonate, born after 37 weeks of gestation to a primigravida mother. The patient was normally delivered, without hypoxia (Apgar score 10-10) and with no visible dysplasias. The birth weight was 3252 g, with a height of 51 cm and a head circumference of 34 cm. There was no conflict between blood types and Rh factors; there was no history of heritable diseases in the family.

During the first day after birth, the newborn started to be fed her mother’s breast milk. However, on the second day of life, signs of jaundice and lethargy began to appear, the newborn was then transferred from a small town hospital to LUHS Kaunas Clinics. There was an increase in the patient’s blood levels of total bilirubin (from 281 to 402.88 µmol/L) and conjugated bilirubin (from 33.84 to 44.59 µmol/L); glycemia (4.3 mmol/L) and C-reactive protein (CRP) (1.33 mg/L) were within normal range. The patient was treated with phototherapy, however, jaundice became more prominent with time. On the seventh day of life, the patient’s skin became strongly bronze-colored, signs of gastrointestinal bleeding began to appear, in conjunction with hepatomegaly and acute liver failure. Blood clotting was severely compromised (Owren’s *Stago* Prothrombin Assay (SPA) = 5%, international normalized ratio (INR) = 8.2), concentrations of both total and conjugated bilirubin remained high (394.47 µmol/L and 41.63 µmol/L respectively), while liver enzymes were within normal range. (In Lithuania and Scandinavia, Owren’s SPA is more commonly used than prothrombin time.) The patient was gradually losing weight, over 7 days she had lost ~15% of her body weight at the time of birth, even though at the time she was being regularly given her mother’s breast milk via a nasogastric (NG) tube.

Over the period of the next two days, the patient’s condition continued to deteriorate, excessive bleeding from puncture sites was observed, together with signs of progressing liver failure. The patient still retained a strong bronze color of the skin, and signs of lethargy were still being observed. A metabolic disease was suspected; the enteral feeding of breast milk was discontinued and the patient was started on parenteral nutrition. At that time, the biochemical blood profile showed decreasing levels of total protein (from 50.01 to 32.10 mg/L) and albumin (from 23 to 18 mg/L), clotting parameters remained abnormal (Owren’s SPA = 5%, INR = 8.2), CRP was not substantially elevated (35.4 mg/L), complete blood count indicated leukopenia, neutropenia and thrombocytopenia. However, the biochemical blood profile also revealed slightly decreasing concentrations of ammonia (from 100 to 68 µmol/L) and total bilirubin (from 265.46 to 185.96 µmol/L), while showing slightly increasing levels of conjugated bilirubin (from 45.73 to 57.33 µmol/L). The patient was diagnosed with sepsis (blood cultures grew *E. coli*, sensitive to cefuroxime and gentamicin), and antibacterial treatment was started. With continuing antibacterial and symptom-alleviating treatment, as well as the discontinuation of breast milk, the patient’s condition was stabilized. Many possible causes of newborn hepatitis were rejected, including infections, hematological and other metabolic disorders.

On the patient’s ninth day of life, information was received from the Vilnius University Hospital Center for Medical Genetics (responsible for administering the newborn screening program) that there is a strong reason to believe that the patient is suffering from galactosemia (galactose conc. 236.29 mg/dL). The patient was started on an amino acid-based formula without lactose and galactose *Neocate Syneo* via a nasogastric tube, while also continuing partial parenteral nutrition. Her condition started rapidly improving, body weight started growing, biochemical blood profile showed improvement, and signs of liver failure disappeared. The galactosemia diagnosis was confirmed via the Sanger sequencing method—a novel, likely pathogenic, homozygous variant in the *GALT* gene NM_000155.4:c.305T>C (p.Leu102Pro) was identified.

Patient 2 was a female neonate, born after 40 weeks of gestation to a primigravida mother. The patient was normally delivered, without hypoxia (Apgar score 10-10) and with no visible dysplasias. Body mass at birth was 3400 g, 52 cm in height, with a head circumference of 36 cm. It is known from family history that the biological mother had a mental illness, and the father was suffering from diabetes and chronic alcoholism. There was no conflict between blood types and Rh factors. The patient was being fed her mother’s breast milk by bottle. Even though the newborn was eagerly consuming breast milk, she lost 12% of her body weight over a 2-day period. On the third day of life, symptoms of jaundice and lethargy began to appear—the patient was transferred from a small town hospital to the LUHS Kaunas Clinics hospital for all further treatment.

From the biochemical blood profile (performed at the LUHS hospital), an elevated concentration of total bilirubin was observed (304 µmol/L), with unconjugated bilirubin being the dominant form (16 µmol/L of conjugated bilirubin). Over the following 4 days, while applying phototherapy, jaundice was becoming more prominent, the patient developed hepatomegaly, body weight continued to decline (the patient lost 16% of her body weight at birth in total). After that period of 4 days, even larger concentrations of total bilirubin (509.7 µmol/L) and conjugated bilirubin (50 µmol/L) were measured in the patient’s blood. The patient had increased levels of liver enzymes (aspartate transaminase (AST)—128 IU/L, alanine transaminase (ALT)—150 IU/L), while gamma-glutamyltransferase (GGT) and alkaline phosphatase (ALP) remained within normal range. Blood clotting factors (Owren’s SPA = 70%), albumin (29 g/L), total blood protein (45 g/L), glycemia (3.97 mmol/L) and CRP (5 mg/L) remained within normal range. Due to ongoing lethargy, neurosonography was performed, signs of ventriculomegaly, calcinates, and subependemic cysts were found, however, every possible heritable infection (TORCH complex) was ruled out.

On the patient’s ninth day of life information was received from the Vilnius University Hospital Center for Medical Genetics (administering the newborn screening program) that the patient was suspected of having galactosemia (galactose conc. 210.12 mg/dL). Thus, the patient was no longer given breast milk, and instead was switched over to an amino acid-based formula without lactose and galactose *Neocate LCP*. After the patient stopped receiving breast milk, her condition started rapidly improving. After 3 days, the patient started gaining weight, signs of jaundice disappeared and the levels of biochemical blood indicators had normalized (both total bilirubin and its fractions, liver enzymes). The diagnosis of galactosemia was confirmed via Sanger sequencing—the patient was homozygous for the pathogenic *GALT* variant NM_000155.3(*GALT*):c.329−2A>C.

As the patients grew older, their psychomotor development was normal; subsequent biochemical blood profile metrics remained within normal range. The summary and comparison of the clinical symptoms and laboratory test results for each patient is outlined in Table 1.

Informed consent was obtained from the parents/guardians of both patients, who agreed for case records to be made available for scientific publication.

## 3. Discussion

Both of our patients right after birth appeared to be healthy, with no signs of dysplasia. No clinical symptoms were exhibited during the first day after birth by both patients, even though they were being fed their mother’s breast milk. We know from the literature that even though newborns suffering from galactosemia appear to be healthy upon visual examination following birth, after they begin to be fed either breast milk or a cow’s milk-based formula, they develop life-threatening symptoms, including damage to the liver, kidneys and the central nervous system [2].

The symptoms of classic galactosemia usually appear in the first week after galactose is ingested [5,6]. In the case of our two patients, the disease symptoms started to appear on the second to third days of life, when they started receiving breast milk. The early symptoms of galactosemia are non-specific, and include vomiting, diarrhea, failure to thrive, weight loss, hypotonia and lethargy. Later, conditions such as an enlarged liver and cataracts may appear, the patient may develop sepsis (most often caused by *E. coli*), which is one of the main causes of death in patients with galactosemia [3]. The main symptoms of both of these newborns were jaundice, hepatomegaly, a noticeable loss of body weight during the first week of life, lethargy and listlessness; however, they did not exhibit vomiting or diarrhea. The general condition of Patient 1 was much more serious due to the development of acute liver failure and *E. coli*-related sepsis. One possible cause of many galactosemia patients developing galactosemia-related sepsis is the negative effect of high levels of galactose and its metabolites on leukocyte antibacterial activity [11]. Furthermore, cataracts were not diagnosed in either one of our patients. Some authors note that cataracts are found in 14–30% of patients with galactosemia [5,10].

Galactosemia can be suspected based on the previously mentioned non-specific symptoms, however, the diagnosis is only confirmed after performing a GALT enzymatic activity assay and/or after a pathogenic allele in the *GALT* gene is identified [12]. A different mutation in the *GALT* gene was identified for each of our two patients. The condition of Patient 1, who was homozygous for *GALT* gene variant c.305T>C (p.Leu102Pro), was much more serious (skin bronze in color, indicating very high bilirubin levels, liver failure, bacterial sepsis) than that of Patient 2. More than 300 pathogenic *GALT* variants causing galatosemia can be found in the literature, and thus the clinical symptoms and complications, as well as their frequency, vary [3]. The *GALT* gene variant c.305T>C (p.Leu102Pro) is a novel mutation as it has not yet been described in the literature. Based on our clinical observations, it appears that this mutation might be responsible for a more severe progression of the disease.

The pathogenicity of the NM_000155.4:c.305T>C (p.Leu102Pro) gene variant was defined according to the American College of Medical Genetics and Genomics (ACMG) guidelines [13]. The variant is in the hotspot gene region (PM1, moderate). The variant was not found in gnomAD exomes (good gnomAD exomes coverage = 98.0) and in gnomAD genomes (good gnomAD genomes coverage = 31.5) (PM2, moderate); 206 out of 210 non-VUS missense variants in the *GALT* gene were pathogenic = 98.1%, which is more than the threshold value of 51.0%, and 324 out of 517 clinically reported variants in the *GALT* gene are pathogenic = 62.7%, which was more than the threshold value of 12.0% (PP2, supporting) [14]. The pathogenic computational verdict was based on 11 pathogenic predictions from BayesDel_addAF, DEOGEN2, EIGEN, FATHMM-MKL, LIST-S2, M-CAP, MVP, MutationAssessor, MutationTaster, REVEL and SIFT, in contrast to 2 benign predictions from DANN and PrimateAI (PP3, supporting). Thus, the NM_000155.4:c.305T>C (p.Leu102Pro) gene variant was predicted to be likely pathogenic.

The condition of newborns with galactosemia improves when they stop receiving breast milk (or cow’s milk-based formula) [2,10]. Some studies show that jaundice, hepatosplenomegaly and liver enzymes return to their normal levels after a period of 1 month, on average [10]. The general condition of our patients started improving as early as 2–3 days after they stopped receiving breast milk. Jaundice subsided and liver enzymes returned to normal levels after 3 days for Patient 2 and 2 weeks for Patient 1, which was mostly based on the severity of their symptoms. Hepatomegaly was no longer observed in our two patients after approximately 1 month.

Galactosemia is a life-threatening condition resulting in the build-up of toxic galactose metabolites in many tissues and organs of newborns. If immediate treatment is provided via a lactose- and galactose-free diet, an acute toxic state is avoided and the resulting damage to various body tissues is reduced. This genetic condition ought to be diagnosed quickly and that could be achieved only by universal newborn screening, which has already been practiced in Lithuania for the past 5 years.

The Galactosemia Network (GalNet) recommends that those suffering from galactosemia stay on a life-long diet restricting the intake of foods containing lactose, galactose, or both [12]. If left untreated, galatosemia results in damage to multiple organ systems (liver failure, tubulopathy, early cataracts, developmental disorders, ataxia, etc.) as well as the death of the newborn/infant affected due to long-term effects of toxic galactose metabolites [6].

Our case analysis is a clinical example, which ought to be familiar to every physician—in the case of similar symptoms as described above, there should be a rapid response to the newborn’s condition in terms of suspecting and confirming the diagnosis of galactosemia in order to avoid adverse complications. Whenever a newborn is displaying jaundice, sepsis and such signs of liver failure as coagulopathy and hypoalbuminemia, it is essential to consider galactosemia as a possible (and likely) diagnosis.

## 4. Conclusions

The first symptoms of galactosemia appear in newborns in their first days of life after being fed breast milk (or formula containing lactose). The main clinical symptoms are jaundice (which is the first symptom to appear), significant weight loss, lethargy, and hepatomegaly. A likely pathogenic *GALT* gene variant c.305T>C (p.Leu102Pro) was identified and we believe it could be responsible for a more severe course of the disease, although further study is needed to confirm this. It is very important to suspect and diagnose galactosemia as early in its course as possible, and introduce lactose-free formula into the patient’s diet. Wide-scale newborn screening and genetic testing are particularly crucial for the early detection of the disease.

## Figures and Tables

**Table 1:** Clinical symptoms and laboratory test results for two galactosemia patients in Lithuania, compared to the frequency of symptoms among galactosemia patients in the literature.

	Patient 1	Patient 2	Frequency of Symptoms (% of Cases) [3]
Age (at which first symptoms started to appear)	Second day of life	Third day of life	
Gender	Female	Female	
Family history	No genetic diseases	Mother was diagnosed with mental illness, father had diabetes, chronic alcoholism	
Clinical symptoms			
Lack of weight gain, weight loss	Yes	Yes	29
Jaundice	Yes	Yes	74
Hepatomegaly	Yes	Yes	43
Lethargy	Yes	Yes	16
Feeding problems	Yes	Yes	23
Acute liver failure	Yes	No	9
Sepsis (Gram-negative)	Yes	No	10
Cataracts	No	No	14–30 [5,10]
Laboratory test results			
Hyperbilirubinemia, with unconjugated bilirubin predominating	Yes	Yes	10
*GALT* gene sequencing results	Individual homozygous for NM_000155.4: c.(305T>C); (305T>C) (p.Leu102Pro)	Individual homozygous for NM_000155.3: c.(329-2A>C]; (329-2A>C)	
